# Assessment of Growth Disturbance in Japanese Children with IBD

**DOI:** 10.1155/2010/958915

**Published:** 2010-05-05

**Authors:** Tetsuo Shono, Mayuko Kato, Yo Aoyagi, Hidenori Haruna, Tohru Fujii, Takahiro Kudo, Yoshikazu Ohtsuka, Toshiaki Shimizu

**Affiliations:** Department of Pediatrics, Juntendo University School of Medicine, 2-1-1, Hongo, Bunkyo-ku, Tokyo 113-8421, Japan

## Abstract

In Japan, there is as yet no report on growth retardation in children with IBD. We therefore investigated the cause of growth retardation in Japanese children with IBD. We investigated the height, body weight, serum levels of albumin, IGF-I, CRP, and cytokines, and the amount of corticosteroid administered in children with Crohn's disease (CD, *n* = 15) and ulcerative colitis (UC, *n* = 18). Our results suggest that growth retardation is already present before the initial visit in children with CD, and chronic inflammation may be responsible this growth disturbance. Moreover, the amount of PSL used may contribute to growth retardation by decreasing the serum levels of IGF-I in children with IBD.

## 1. Introduction

Inflammatory bowel disease (IBD), including Crohn's disease (CD) and ulcerative colitis (UC), is a noninvasive chronic inflammatory digestive tract disorder. Recently, there has been an increase in the number of children affected with IBD, as well as a tendency to affect younger patients, as described in Japanese pediatric reports [1]. Although the cause of IBD remains incompletely understood, this disorder is thought to be caused by the intricate involvement of immunological abnormalities, genetic anomalies, and environmental factors [2, 3]. Sawczenko and Sandhu [4] reported that while abdominal pain, diarrhea, and bloody stool have been demonstrated as symptoms of IBD, child IBD differs from adult IBD in that it is associated with short stature due to growth retardation. With children, prior to the development of typical symptoms manifesting in the form of diarrhea and bloody stool, an insufficient increase in body weight is often found and growth retardation can be observed when growth is plotted on a chart. Motil et al. [5] reported that growth retardation occurs more in children with CD than in those with UC. 

The mechanisms underlying insufficient increase in body weight and growth retardation are reportedly attributed to a reduction in energy intake, incomplete gastrointestinal tract absorption, an increase in energy requirements, growth regulation by inflammatory cytokines, and long-term use of therapeutic steroids [6–11]. Particularly in cases involving children that are difficult to treat, growth retardation associated with long-term administration of steroids is a major problem [8]. There are reports such as suppression of chondrocyte proliferation in the growth plate and suppression of proteoglycan composition as growth-inhibiting mechanisms of steroids, because proliferation of growth plate chondrocytes results in the bone growth [12, 13]. However, details of these mechanisms remain unclear.

At present, there is no long-term observational study monitoring growth in Japanese children with IBD prior to treatment and subsequent to steroid therapy. We therefore investigated the growth retardation in Japanese children with CD and UC and explored the mechanism underlying such growth retardation, including malnutrition, chronic inflammation, and steroid administration. 

## 2. Methods

### 2.1. Subjects

We performed this study in compliance with the Helsinki Declaration of the World Medical Association. Additionally, the study was approved by the Clinical Research Ethics Committee of the Juntendo University Hospital and written parental consent was obtained before inclusion. 

We obtained informed consent and enrolled 15 children diagnosed with CD (male/female: 11/4) and 18 children diagnosed with UC (male/female: 8/10) among children with ages ranging from 9 months to 15 years and 8 months who were our patients at the Department of Pediatrics, Juntendo University School of Medicine or affiliated facilities, and whom we were able to observe continuously from the start of treatment up to their follow-up examinations (Table 1).

### 2.2. Procedures

 We collected data of the children from their records which included height and weight at 12 months and 6 months before the initial IBD diagnosis, at the initial assessment, and at 6 and 12 months following the start of treatment subsequent to the initial assessment. From the collected data, a growth chart was generated using HONETARO ver.5 software (TGL Co., Osaka, Japan). From the obtained data, height curve SDS and % standard weight were calculated. Growth rate that was shown as a change in height SDS during 1 year was also determined using POCKET GROWTH CHECKER III ver. 1.0 (Ultmarc Co., Tokyo, Japan). IGF-I, IL-6, TNF-*α*, and TGF-*β* were measured by enzyme-linked immunosorbent assay (ELISA) using commercially available assay kits (Fujirebio, Tokyo, Japan). The CD remission period was defined as having a CD Activity Index (CDAI) assessed by International Organization for the Study of IBD [14] of 0 or 1, CRP-negative, and normal erythrocyte sedimentation. The UC remission period was defined as the absence of fever, abdominal pain, and bloody stool, with the stool frequency and fecal characteristics returning to pre-IBD conditions, testing negative for CRP and with normal erythrocyte sedimentation. Severity of children with CD and UC was assessed by the method of Turner et al. [15] and Truelove and Witts [16], respectively.

We used the Wilcoxon *t*-test, Mann-Whitney *U*-test, and Spearman rank analysis for statistical analysis.

## 3. Results

There was no significant difference in age at the initial visit between children with UC and those with CD; however, children with CD showed a significant longer period from the onset to the initial visit than those with UC (*P* < .05).

**Figure 1:**
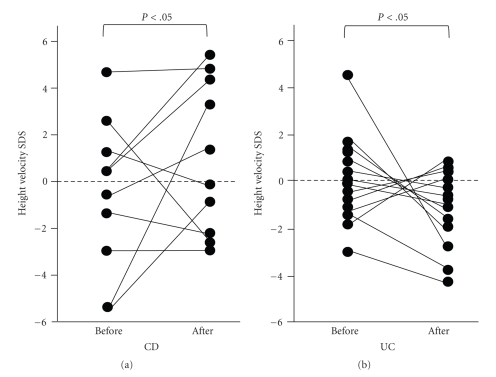
Comparison of growth rate SDS between children with CD and those with UC during the 1-year period before the initial assessment and the 1-year period subsequent to the initial assessment. before: 1 year to initial visit; after: from initial visit to 1 year later.

**Figure 2:**
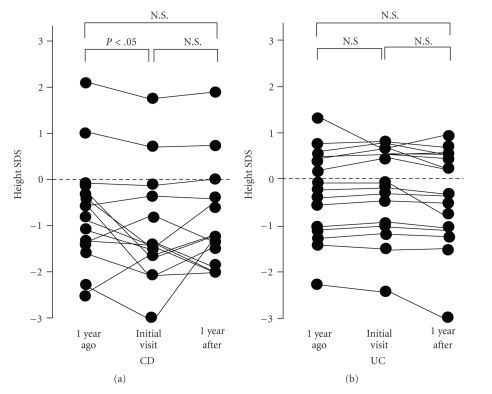
Changes in height SDS in children with CD and those with UC.

There was a significant increase in the growth rate SDS in children with CD (*P* < .05) but a significant decrease in children with UC (*P* < .05) when we compared the growth rate during the 1-year period before the start of treatment with the growth rate during the 1-year period subsequent to treatment (Figure 1). Compared with the 1-year period before the start of treatment, height SDS in children with CD at diagnosis was significantly lower (*P* < .05); however, there was no significant difference between the pretreatment level and the level 1 year subsequent to the start of treatment. On the other hand, in children with UC, there was no significant difference in height SDS when comparisons were made between values obtained 1 year before the start of treatment, at diagnosis, and 1 year subsequent to the start of treatment (Figure 2).

**Figure 3:**
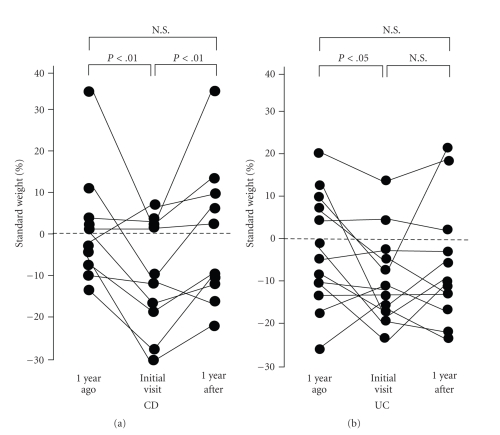
Changes in % standard weight in children with CD and those with UC.

Compared with the 1-year period before the start of treatment, % standard weight in the children with CD at diagnosis was significantly lower (*P* < .01) but showed a significant increase 1 year subsequent to the start of treatment (*P* < .01). On the other hand, there was no significant difference between the pretreatment and posttreatment levels. In subjects with UC, compared with the 1-year period before starting treatment, % standard weight at diagnosis was found to be significantly lower (*P* < .05), and although posttreatment levels tended to be elevated, we found no significant difference between the pretreatment and posttreatment levels (Figure 3).

We also compared serum levels of albumin, IGF-1, CRP, IL-6, TNF-*α*, and TGF-*β*, parameters associated with growth, in subjects with CD and those with UC at the initial assessment and 1 year after the start of treatment. Although we found that the CRP level was significantly (*P* < .05) elevated in subjects with CD compared with those with UC at the initial assessment, there were no significant differences between any of the other parameters (Table 2). On the other hand, we found no significant differences in any of these parameters between subjects with CD and those with UC 1 year after starting treatment.

Furthermore, we compared the amount of prednisolone (PSL) used (per kg body weight) and the length of remission both 6 months and 1 year after the start of treatment between children with CD and those with UC; however, there were no significant differences (Table 3).

**Figure 4:**
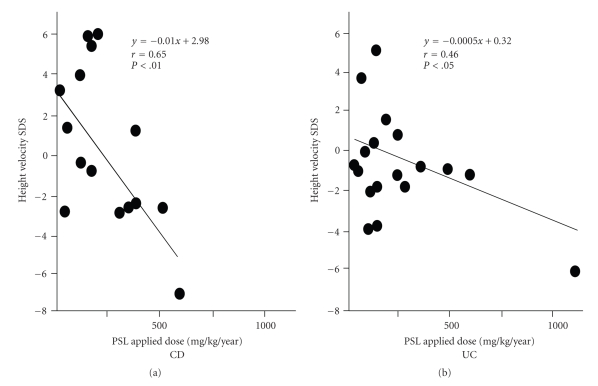
Correlation between growth rate SDS and the amount of PSL administered in children with CD and those with UC from the start of treatment to 1 year posttreatment.

**Figure 5:**
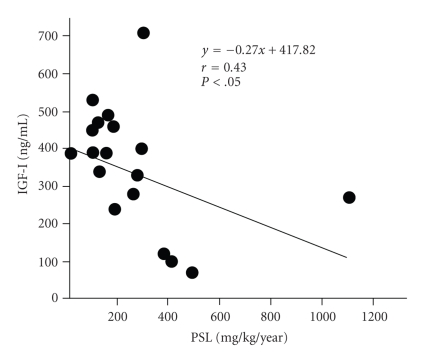
Correlation between serum IGF-I level and the amount of PSL administered in children with IBD in the 1-year period following the start of treatment.

We found a significant negative correlation in both groups (CD group, *P* < .01; UC group, *P* < .05) when we compared the correlation between the amount of PSL used and growth rate 1 year after the start of treatment (Figure 4). We also found a negative correlation between the amount of PSL used and IGF-1 level in children with IBD (Figure 5).

## 4. Discussion

The present results indicate that children diagnosed with IBD suffered from a significant decrease in body weight, both in children with CD and UC at the start of treatment compared with their weight 1 year before the start of treatment. Although there was a significant decrease in height in the children with CD at the start of treatment compared with their height 1 year earlier, there was no corresponding decrease among the children with UC. Moreover, a comparison of growth rate 1 year earlier and 1 year after starting treatment showed a significant increase in children with CD, but a significant decrease in those with UC. These results are consistent with previous results showing that in children with IBD, growth is impaired in those with CD compared with those with UC. Langholz et al. [17] reported growth retardation in some subjects in children with CD compared with children with UC. Prieto et al. [18] reported that in 28 children with IBD, growth retardation occurred with high frequency in children with CD compared with children with UC. Treatment, including the use of corticosteroid, is thought to improve this growth impairment. In our study, actual growth retardation at the time of diagnosis, height <−2.0 SDS was detected 20% of CD patients and 10% of UC patients. On the other hand, Hildebrand et al. [19] reported that height <−2.0 SDS was detected 13% of CD patients and 3% of UC patients. These findings suggest that both CD and UC children in Japan show more frequent growth retardation than those in Sweden. 

In order to determine the cause of the difference in growth impairment between children with CD and those with UC, particularly in terms of increases in height, we compared nutritional parameters, such as albumin and IGF-I, cytokines and inflammatory markers, including CRP, IL-6, and TNF-*α* between children with CD and those with UC. These factors are considered to be related to growth before treatment. We found that only the CRP level in the children with CD was significantly increased. In several previous reports, the CRP level has been shown to correlate with the severity of CD, and has also been reported to be an important indicator of growth retardation [20–24]. Since there was no significant difference in the levels of albumin and IGF-I before the start of treatment in either group, as well as in body weight in both groups, growth impairment before the start of treatment might not be caused by nutritional problems. On the other hand, serum CRP level before treatment was found to be significantly elevated in children with CD, although there were no significant differences in the levels of IL-6 and TNF*α*, indicating that growth impairment before the start of treatment in children with CD might be affected by chronic inflammation. Although not indicated in the obtained data, there was no significant difference in growth rate between children with CD having small intestinal lesions and those having no lesions. These results also suggest that nutritional impairment is not the primary cause of growth impairment in children with CD. Inflammatory markers, including CRP and IL-6, have a correlation to the disease activity. These markers were significantly increased on admission compared to 1 year after. There is, however, no significant correlation between levels of CRP and IL-6 and disease activity.

The amount of steroid used is an extremely important factor affecting post-treatment growth [25]. In the present study, there was no significant difference in the amount of PSL used and the remission period between children with CD and those with UC following the start of treatment at 6 months and 1 year. On the other hand, growth rate and the amount of PSL used showed a negative correlation in children with CD and UC, which may indicate that PSL contributes to growth impairment in IBD children. The reason for the significant difference in growth impairment between children with CD and those with UC after the treatment is that while growth impairment due to chronic inflammation in children with CD occurs prior to treatment, this condition is reversed by anti-inflammatory treatment following the start of PSL therapy. The decreased growth rate in children with UC might be due to the side effect of PSL, because growth rate of children with UC was decreased after the start of PSL treatment. 

Some reports suggest that the mechanism underlying the growth impairment induced by corticosteroid is an IGF-I-mediated mechanism acting on the chondrocytes of the growth plate in a manner that reduces skeletal metabolism [12, 26]. A previous report which investigated the association between the administration of exogenous steroids and IGF-1 has demonstrated that these steroids cause impairment of the IGF axis and a decrease in the level of IGF-binding protein [27]. Although we conducted the present study without considering pubertal age, the negative correlation of IGF-1 level and the amount of steroid used in children with IBD led us to consider that one mechanism underlying the growth impairment induced by corticosteroid in children with IBD is mediated by the decreased serum level of IGF-I. Although, there was no significant correlation between pretreatment IGF-I level and growth rate in our study, a previous study showed that inflammatory cytokines induced a decrease in IGF-I level [28]. Recent study by MacRae et al. [29] indicated that dexamethasone and IL-1*β* might primarily inhibit IGF-1-induced bone growth. Further investigation should be performed to clarify the relationship among IGF-I, cytokines, and corticosteroids in growth disturbance of children with IBD. Moreover we should evaluate their pubertal stage that is known to be of importance in growth velocity to carefully investigate growth disturbance in children with IBD.

In conclusion, we investigated growth impairment in Japanese children with IBD. Chronic inflammation was considered to be responsible for growth impairment in children with CD before the start of treatment; however, subsequent to treatment, the primary cause was believed to depend on the amount of corticosteroid used in children with IBD. On the other hand, the anti-inflammatory effects of corticosteroids might be necessary to suppress growth retardation in children with CD.

## Figures and Tables

**Table 1:** Profile of children with CD (*n* = 15) and those with UC (*n* = 18) (mean ± SD).

	CD		UC	
*n*(M/F)	15(11/4)		18(8/10)	N.S.
Average age at the initial visit (year)	9.8 ± 5.1		10.9 ± 4.0	N.S.
Period of time until the initial visit (week)	28.3 ± 27.4		10.2 ± 9.7	*P* < .05

Type	I	1 case	R	3 cases
	IC	7 cases	L	5 cases
	C	7 cases	T	10 cases
Severity	Mean PCDAI		Mild	3 cases
	36.2 ± 20.0		Moderate	9 cases
			Severe	6 cases

Type I: small intestine; Type IC: small intestine/Colon; Type C: colon.

Type R: rectum; Type L: left colon; Type T: total colon.

PCDAI: Pediatric Crohn's Disease Activity Index [13].

UC severity [14].

**Table 2:** Comparison of various parameters at initial assessment and 1 year after the start of treatment in children with CD and those with UC (mean ± SD).

	Admission			1 year after		
	CD	UC		CD	UC	
Alb (g/dL)	3.51 ± 0.57	3.67 ± 0.65	N.S.	4.36 ± 0.33	4.24 ± 0.39	N.S.
IGF-I (ng/mL)	270.9 ± 219.6	231.8 ± 166.1	N.S.	356.7 ± 164.7	350.2 ± 164.3	N.S.
CRP (mg/dL)	3.16 ± 3.20	1.35 ± 2.59	*P* < .05	0.17 ± 0.29	0.10 ± 0.11	N.S.
IL-6 (pg/mL)	9.74 ± 8.34	11.24 ± 23.69	N.S.	2.93 ± 2.20	2.28 ± 3.73	N.S.
TNF-*α* (pg/mL)	0.76 ± 1.17	0.98 ± 2.19	N.S.	0.66 ± 0.96	1.02 ± 1.25	N.S.
TGF-*β* (ng/mL)	52.71 ± 7.36	47.51 ± 14.10	N.S.	47.80 ± 5.13	44.68 ± 12.89	N.S.

**Table 3:** Comparison of total dose of PSL and remission rates between children with CD and those with UC 6 months and 1 year after the start of treatment (mean ± SD).

	6 months after			1 year after		
	CD	UC		CD	UC	
Total dosage of prednisolone (mg/kg)	116.4 ± 132.5	207.8 ± 220.6	N.S.	180.4 ± 186.5	273.5 ± 282.9	N.S.
Remission rate (%)	54.08 ± 25.92	52.76 ± 28.48	N.S.	61.83 ± 30.60	65.94 ± 19.35	N.S.

## References

[1] Lakatos L, Lakatos PL (2007). Changes in the epidemiology of inflammatory bowel diseases. *Orvosi Hetilap*.

[2] Shih DQ, Targan SR, McGovern D (2008). Recent advances in IBD pathogenesis: genetics and immunobiology. *Current Gastroenterology Reports*.

[3] Lakatos PL (2009). Environmental fantors affecting inflammatory bowel disease: have we meda progress?. *Digestive Diseases*.

[4] Sawczenko A, Sandhu BK (2003). Presenting features of inflammatory bowel disease in Great Britain and Ireland. *Archives of Disease in Childhood*.

[5] Motil KJ, Grand RJ, Davis-Kraft L, Ferlic LL, Smith EO (1993). Growth failure in children with inflammatory bowel disease: a prospective study. *Gastroenterology*.

[6] Oliva MM, Lake AM (1996). Nutritional considerations and management of the child with inflammatory bowel disease. *Nutrition*.

[7] Kappelman MD, Bousvaros A (2008). Nutritional concerns in pediatric inflammatory bowel disease patients. *Molecular Nutrition and Food Research*.

[8] Ballinger A (2002). Fundamental mechanisms of growth failure in inflammatory bowel disease. *Hormone Research*.

[9] Ballinger AB, Camacho-Hübner C, Croft NM (2001). Growth failure and intestinal inflammation. *QJM*.

[10] MacRae VE, Wong SC, Farquharson C, Ahmed SF (2006). Cytokine actions in growth disorders associated with pediatric chronic inflammatory diseases. *International Journal of Molecular Medicine*.

[11] Büller HA (1997). Problems in diagnosis of IBD in children. *Netherlands Journal of Medicine*.

[12] Owen HC, Miner JN, Ahmed SF, Farquharson C (2007). The growth plate sparing effects of the selective glucocorticoid receptor modulator, AL-438. *Molecular and Cellular Endocrinology*.

[13] Stratakis CA (2006). Cortisol and growth hormone: clinical implications of a complex, dynamic relationship. *Pediatric Endocrinology Reviews*.

[14] De Dombal FT, Softley A (1987). IOIBD report no. 1: observer variation in calculating indices of severity and activity in Crohn’s disease. *Gut*.

[15] Turner D, Hyams J, Markowitz J (2009). Appraisal of the pediatric ulcerative colitis activity index (PUCAI). *Inflammatory Bowel Diseases*.

[16] Truelove SC, Witts LJ (1955). Cortisone in ulcerative colitis; final report on a therapeutic trial. *British Medical Journal*.

[17] Langholz E, Munkholm P, Krasilnikoff PA, Binder V (1998). Inflammatory bowel diseases in children. *Ugeskrift Laeger*.

[18] Prieto Bozano G, Carrasco Gandía S, Lama More R, Miralles Adarraga T, Polanco Allue I (1991). Inflammatory bowel disease in the child. *Anales Espanoles de Pediatria*.

[19] Hildebrand H, Karlberg J, Kristiansson B (1994). Longitudinal growth in children and adolescents with inflammatory bowel disease. *Journal of Pediatric Gastroenterology and Nutrition*.

[20] De Benedetti F, Meazza C, Martini A (2002). Role of interleukin-6 in growth failure: an animal model. *Hormone Research*.

[21] Reimund J-M, Wittersheim C, Dumont S (1996). Increased production of tumour necrosis factor-*α* interleukin-1*β* and interleukin-6 by morphologically normal intestinal biopsies from patients with Crohn’s disease. *Gut*.

[22] Wong SC, MacRae VE, McGrogan P, Ahmed SF (2006). The role of pro-inflammatory cytokines in inflammatory bowel disease growth retardation. *Journal of Pediatric Gastroenterology and Nutrition*.

[23] Eivindson M, Nielsen JN, Grønbaek H, Flyvbjerg A, Hey H (2005). The insulin-like growth factor system and markers of inflammation in adult patients with inflammatory bowel disease. *Hormone Research*.

[24] Beck PL, Podolsky DK (1999). Growth factors in inflammatory bowel disease. *Inflammatory Bowel Diseases*.

[25] Motoyama O, Iitaka K (2007). Final height in children with steroid-sensitive nephrotic syndrome. *Pediatrics International*.

[26] Smink JJ, Koedam JA, Koster JG, van Buul-Offers SC (2002). Dexamethasone-induced growth inhibition of porcine growth plate chondrocytes is accompanied by changes in levels of IGF axis components. *Journal of Endocrinology*.

[27] Smink JJ, Koster JG, Gresnigt MG, Rooman R, Koedam JA, van Buul-Offers SC (2002). IGF and IGF-binding protein expression in the growth plate of normal, dexamethasone-treated and human IGF-II transgenic mice. *Journal of Endocrinology*.

[28] De Benedetti F, Alonzi T, Moretta A (1997). Interleukin 6 causes growth impairment in transgenic mice through a decrease in insulin-like growth factor-I. A model for stunted growth in children with chronic inflammation. *Journal of Clinical Investigation*.

[29] MacRae VE, Ahmed SF, Mushtaq T, Farquharson C (2007). IGF-I signalling in bone growth: inhibitory actions of dexamethasone and IL-1*β*. *Growth Hormone and IGF Research*.

